# Epidemiology of Dengue Disease in Malaysia (2000–2012): A Systematic Literature Review

**DOI:** 10.1371/journal.pntd.0003159

**Published:** 2014-11-06

**Authors:** Abdul Hamid Mohd-Zaki, Jeremy Brett, Ellyana Ismail, Maïna L'Azou

**Affiliations:** 1 Vector Borne Disease Sector, Ministry of Health Malaysia, Kuala Lumpur, Malaysia; 2 Sanofi Pasteur, Singapore Pte. Ltd., Singapore; 3 Sanofi Pasteur, Petaling Jaya, Selangor, Malaysia; 4 Global Epidemiology Department, Sanofi Pasteur, Lyon, France; University of Heidelberg, Germany

## Abstract

A literature survey and analysis was conducted to describe the epidemiology of dengue disease in Malaysia between 2000 and 2012. Published literature was searched for epidemiological studies of dengue disease, using specific search strategies for each electronic database; 237 relevant data sources were identified, 28 of which fulfilled the inclusion criteria. The epidemiology of dengue disease in Malaysia was characterized by a non-linear increase in the number of reported cases from 7,103 in 2000 to 46,171 in 2010, and a shift in the age range predominance from children toward adults. The overall increase in dengue disease was accompanied by a rise in the number, but not the proportion, of severe cases. The dominant circulating dengue virus serotypes changed continually over the decade and differed between states. Several gaps in epidemiological knowledge were identified; in particular, studies of regional differences, age-stratified seroprevalence, and hospital admissions.

**Protocol registration:**

PROSPERO #CRD42012002293

**Author summary**

Dengue disease is a tropical and subtropical mosquito-borne viral illness, and is a major health concern in Malaysia. We conducted this literature analysis and review to describe the epidemiology of dengue disease in Malaysia between 2000 and 2012, to determine the impact of dengue disease on the Malaysian population, and to identify future research priorities. We used well-defined methods to search and identify relevant research, and data were selected according to predetermined inclusion criteria. This long-term review highlights the changing epidemiology of dengue fever in Malaysia. Although the overall incidence has stabilized in recent years, dengue disease remains a public health burden. Our review demonstrates an increased incidence of all forms of dengue disease and a predominantly adult age distribution. Changes in circulating dengue virus serotypes may have implications for the incidence and severity of dengue disease. Increasing levels of rainfall, humidity, temperature, and urbanization have been identified as risk factors for dengue disease outbreak. We believe that the recent improvements to the surveillance system in Malaysia should, if pursued over the next few years, greatly improve our understanding of the burden of dengue fever and enable us to monitor the impact of disease control measures in the future.

## Introduction

Dengue disease, a tropical and subtropical mosquito-borne viral illness, is a major health concern worldwide. A recent disease distribution model estimated that there were 96 million apparent dengue virus (DENV) infections globally in 2010 and that Asian countries, with 67 million apparent infections, bore a disproportionate infectious burden (70%) [1]. Within Asia, the World Health Organization (WHO) Western Pacific Region (WPRO) is considered to be the global epicentre of the disease [2] and Malaysia ranked third among countries in the WPRO in terms of the number of reported cases of dengue disease in the period 1991–2007 [3]. Dengue disease was first reported in Malaysia in the early 1900s [4] and became a public health problem in the 1970s [5]. Significant outbreaks of dengue fever (DF) occurred from 1982, with a gradually increasing pattern of incidence and fatalities [3]. The disease has been endemic since the early 1990s, with yearly and frequent outbreaks thereafter [3], [6]. Over the past few decades, major dengue disease outbreaks occurred in a cyclical pattern of approximately 8 years, involving mainly DENV-1, DENV-2, and DENV-3 serotypes [7]. This cycling correlated with the switching of the predominant DENV serotypes in the population.

Current determination of dengue disease cases in Malaysia is by clinical diagnosis, using the WHO 1997 criteria for DF and dengue haemorrhagic fever (DHF) [8]. There is a standard triage system for diagnosis, monitoring, and management by the physician [9], and control measures are instituted after dengue disease cases have been verified. Case reporting is mandated legally, and vector surveillance is conducted as part of a national surveillance system [10]. Suspected dengue disease cases are notified within 24 hours to the nearest District Health Office for investigation through the National Notifiable Infectious Diseases system. Serotype analysis is undertaken by all Public Health Laboratories. There is no requirement in the reporting regulations for laboratory testing, but 40–50% of cases are confirmed in this way by all hospitals and some health clinics in Malaysia using a passive system [11].

**Figure 1:**
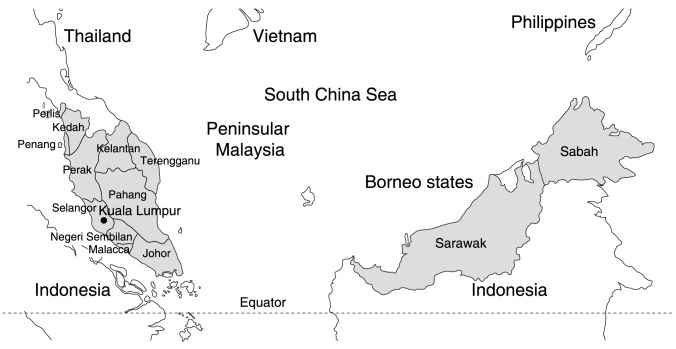
Map of Malaysia. Malaysia is a federation separated into two regions by the South China Sea. There are 11 states and two federal territories on Peninsular Malaysia and two states and one federal territory in East Malaysia. The Peninsular states are divided into districts. On Borneo, the Sabah and Sarawak districts are grouped into divisions.

The population of Malaysia in 2012 was estimated to be 28,855,000 [12]. The main races are Malay (50·4%), Chinese (23·7%), and Indian (7·1%) [13]. Malaysia consists of two geographical regions (Peninsular and Eastern Malaysia, separated by the South China Sea; latitude 2u309 N, longitude 112u309 E) and is divided into 13 states and three federal territories (**Figure 1**). Most people (81%) live in coastal areas and on peninsular Malaysia [13]. Since the mid 1970s, urbanization has increased markedly [14]. The climate is characterized by high average temperatures and rainfall, with only small differences in temperature reported throughout the year. High rainfall patterns follow the monsoon winds, which occur between November and March and from June to September [13].

This article describes the national and regional epidemiology of dengue disease in Malaysia reported in the literature from 2000 to 2012. It aims to identify gaps in epidemiological knowledge and future research needs. Incidence (by age and sex), seroprevalence and serotype distribution, and other relevant epidemiological data are described.

## Methods

The overall methodology, search strategy, and inclusion and exclusion criteria for this literature analysis and review are included in a protocol that was developed by a Literature Review Group (LRG). The protocol was based on the preferred reporting items of systematic reviews and meta-analyses (PRISMA) guidelines [15] and was registered on PROSPERO, an international database of prospectively registered systematic reviews in health and social care managed by the Centre for Reviews and Dissemination, University of York, on 16 July 2012 (http://www.crd.york.ac.uk/prospero/display_record.asp?ID=CRD42012002293).

### Search strategy and selection criteria

The LRG guided the literature analysis process, defined the search strategy, and prepared the protocol and review documents. Search strings for each database were designed with reference to the expanded Medical Subject Headings thesaurus, encompassing the terms ‘dengue’, ‘epidemiology’, and ‘Malaysia. Different search string combinations were used for each electronic database with the aim of increasing the query's sensitivity and specificity.

The protocol directed that only studies published in English during the survey period (1 January 2000 to 23 February 2012) were to be included in the analysis. For databases that did not allow language and/or date limitations, references not meeting these criteria were deleted manually at the first review stage. No limits were imposed by sex, age, and race of study participants or by study type, although single-case reports were excluded, as were studies that only reported data for the period before 1 January 2000. To avoid duplicate publication of data, literature reviews and editorials utilising previously published peer-reviewed data were excluded. Unpublished reports were included if they were identified in one of the sources listed above. Finally, publications not identified by the approved search strategy and unpublished data sources that met the inclusion criteria were included if recommended by members of the LRG.

Searches of published literature were conducted for epidemiological studies of dengue disease between 9 February 2012 and 23 February 2012, in the following databases: PubMed, Excerpta Medica Database (EMBASE), MedLine, WHO Library database (WHOLIS), WPRO, Index Medicus for South-East Asia Region (IMSEAR), and the Malaysian Ministry of Health (MoH) official bulletins. Literature relating to key congresses, grey literature such as lay publications, Dengue Bulletins, and theses, and general internet searches were conducted to complement data gathered in the published literature. General internet searches were limited to the first 50 results.

We utilised an inclusive search strategy to find papers, theses, dissertations, reports and statistical tables, as well as to official web sites and grey materials. The Literature Review Group defined the inclusion/exclusion criteria and guided the search and selection process. Decisions were made by reaching a consensus via teleconferences. It was expected that the resulting articles would be heterogeneous with respect to data selection, and classification of cases, and would not be methodologically comparable. We therefore planned not to perform a meta-analysis.

After removing duplicate citations, the Literature Review Group reviewed the titles and abstracts. The full text any published sources selected was retrieved electronically or in paper form and a second review was performed to make the final selection of relevant sources to include. Sources were reviewed by the Literature Review Group to ensure they complied with the search inclusion and exclusion criteria. In particular, single-case reports and studies conducted before or after the defined search period were excluded, as were publications of duplicate data sets, unless the articles were reporting different outcome measures.

We chose not to exclude articles and other data sources nor formally rank them on the basis of the quality of evidence. Whilst we recognize that assessing study quality can potentially add value to a literature review, the consensus of the Literature Review Group was that given the expected high proportion of surveillance data among the available data sources and the nature of surveillance data (passive reporting of clinically-suspected dengue), such quality assessment would not add value to our review.

The selected data sources were collated and summarized using a data extraction instrument developed as a series of Excel (Microsoft Corp., Redmond, WA) spreadsheets. Data from literature reviews of previously published peer-reviewed studies and pre-2000 data published within the search period were not extracted. The original data sources and the extraction tables were made available to all members of the Literature Review Group for review and analysis. A narrative synthesis of our findings is presented.

### Protocol amendment

A protocol amendment was prepared to allow analysis of data on file at the Malaysian MoH up to July 2012. The Vector Borne Disease Sector of the MoH provided the LRG with additional data on dengue disease epidemiology from 2000 to 2012 on 10 July 2012 [12]. These data were also collated and summarized using the data extraction instrument.

## Results

**Figure 2:**
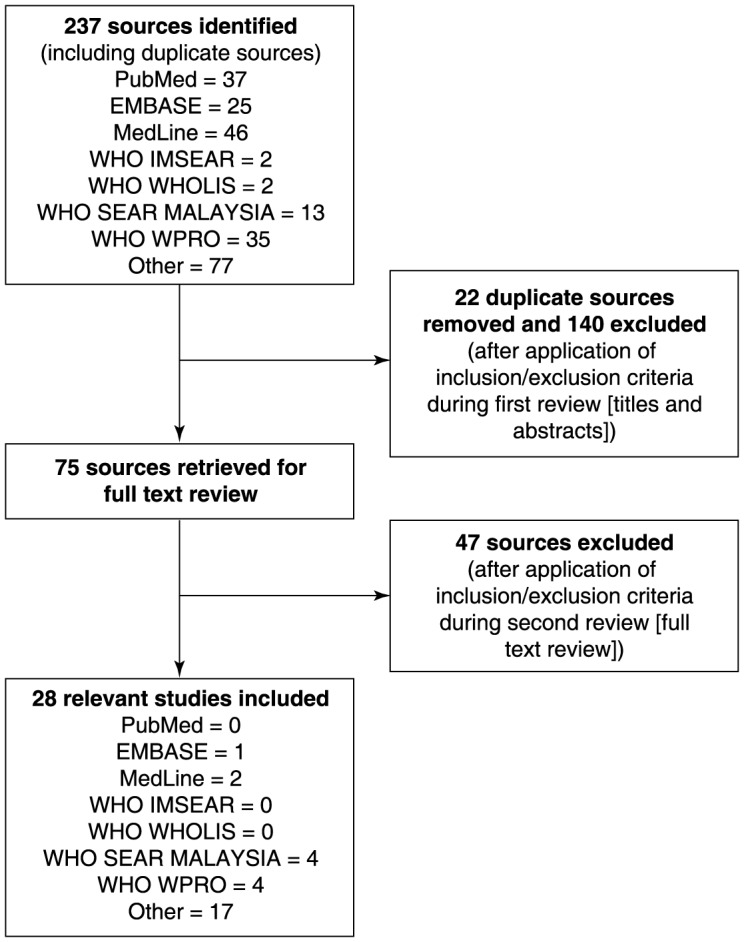
Results of literature search and evaluation of identified studies according to PRISMA. All references identified in the on-line database searches were assigned a unique identification number. Following the removal of duplicates and articles that did not satisfy the inclusion criteria from review of the titles and abstracts, the full papers of the first selection of references were retrieved either electronically or in paper form. A further selection was made based on review of the full text of the articles. EMBASE, Excerpta Medica Database; PRISMA, preferred reporting items of systematic reviews and meta-analyses; WHO IMSEAR, World Health Organization Index Medicus for South-East Asia Region; WHO WHOLIS, World Health Organization Library Database; WHO SEAR MALAYSIA, World Health Organization Regional Office for Southeast Asia Malaysia; WHO WPRO, World Health Organization Western Pacific Region.

This review concentrates on national epidemiological data, including the latest unpublished data received from the Malaysian MoH [12]. Data were collated from several sources [3], [9], [12], . Most of the national epidemiological data for the period 2000–2012 were derived from annual surveys or statistical tables produced by the Malaysian MoH and published by the MoH and the WHO (**Table S1**). These were either found during the initial searches or recommended by members of the LRG to supplement incomplete data presented in the reports. The literature searches identified 237 relevant data sources, 28 of which fulfilled the inclusion criteria for the analysis (**Figure 2** **; Table S1**). Of the 28 sources, there were 14 journal articles that mainly described regional epidemiological data derived from small surveys and studies conducted in individual Malaysian states and regions (**Table S1**). These are reported here briefly as supporting data.

### National epidemiology (Supplementary Tables S2 and S3)

**Figure 3:**
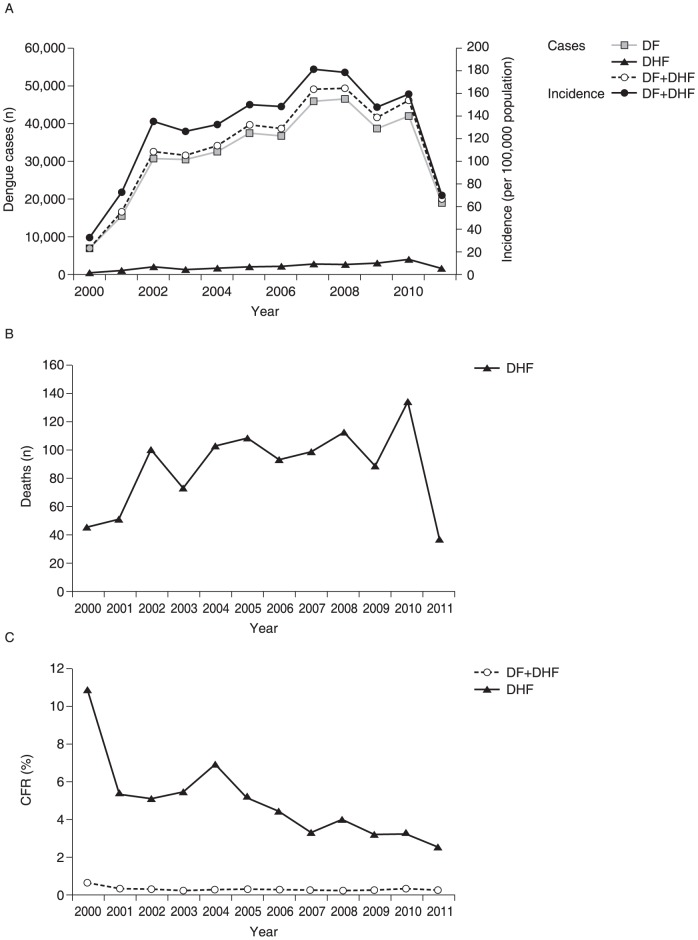
Reported dengue disease cases and deaths in Malaysia 2000–2011 [3], [9], [12], [13], [16]–[23]. (A) Number of reported dengue disease cases (incidence per 100,000 population shown as numbers for the total cases only). (B) Number of reported deaths. (C) CFRs. The total annual number of dengue disease cases reported in Malaysia increased between 2000 and 2010 and decreased in 2011. Between 2000 and 2010, the incidence rate of dengue disease was consistently high (above 125 per 100,000 population). The majority of dengue disease cases were reported as DF and the ratio of DF to DHF cases remained fairly constant. The number of annual deaths from dengue disease increased between 2000 and 2010. However, after 2000 the CFR for DF+DHF remained fairly constant. CFR, case fatality rate; DF, dengue fever; DHF dengue haemorrhagic fever.

The population of Malaysia has risen by 23% since 2000, from 23,495,000 in 2000 to 28,855,000 in 2012 [12]. The total annual number of dengue disease cases reported in Malaysia increased from 7103 in 2000 to 46,171 in 2010, with the incidence rising from 31·6 to 159·7 per 100,000 population. During the period 2002–2010, the incidence rate of dengue disease was consistently high (above 125 per 100,000 population), peaking at 180·0 and 178·0 per 100,000 population in 2007 and 2008, respectively (**Figure 3A**) [3], [9], [12], [13], [16]–[23]. However, fewer cases were reported in 2011 compared with previous years (19,884 cases, incidence 69·6 per 100,000 population) [12]. Overall, approximately 50% of dengue disease cases in Malaysia were confirmed by laboratory analysis. This percentage varied from 39·6% to 53·0% in the period 2000–2007, the last year for which data were available [9].

The majority of dengue disease cases were reported as DF, rising from 6692 in 2000 to 42,140 in 2010, the numbers closely matching those of the total cases. The total number of annual DHF cases also increased, from 411 in 2000 to 4031 in 2010 (**Figure 3A**). The ratio of DF to DHF cases remained fairly constant, being 16·3∶1 in 2000 and 10·5∶1 in 2010 [3], [9], [12], [13], [16]–[23]. Again, there were fewer cases of DF and DHF in 2011 compared with previous years (DF 18,466 cases; DHF 1418 cases).

The annual number of deaths from dengue disease increased from 45 in 2000 to 134 in 2010, although the case fatality rate (CFR) remained constant at 0·2–0·3%, with 0·63% reported for the year 2000 (**Figure 3B, 3C**) [3], [9], [12], [21]. The CFR for DHF was relatively high in the year 2000 (10·9%) and then ranged between 2·5 and 6·9% from 2001 until 2011, with an apparent decline from 2006 until 2011 (**Figure 3C**).

### Age, sex, and racial distribution

The data we collected indicate that number of reported cases of dengue disease declined in children but was more stable in adults during the review period. Similar age distributions were reported for both males and females and in the Malay and Indian racial groups, with the highest proportion of dengue disease cases occurred in people aged 10–29 years [12], [16]. The number of deaths due to dengue disease in children (0–14 years) declined markedly up to 2007 as did the CFR (from 1·3% to 0·17%), while the number in adults (aged ≥15 years) increased slightly and the CFR changed little [3], [9].

National data published by the WHO for the period 2000–2008 demonstrated that there was a predominance of males with dengue disease (55–62%). This relationship was confirmed by the proportion of males with dengue disease in all years being significantly greater (p<0·001) than the proportion of males in the population (50·8% in 2000) [16]. The same pattern was reported in MoH data covering 2000–2011, with the proportion of males with reported dengue disease ranging from 54·7% to 61·5% [12].

Data reported for the period 2000–2011 among the different racial groups living in Malaysia demonstrated that the distribution of dengue disease by race broadly reflected the racial distribution of the country as a whole, except in the years 2003 and 2004 [12], [13].

### Other epidemiological features (socio-demographic and seasonal patterns)

A case–control study conducted in Johor Bahru in south peninsular Malaysia demonstrated that the only socio-demographic factors linked significantly to dengue disease were unmarried status (p = 0.006), not wearing long-sleeved clothes (p = 0.047), and not having window screens (p = 0.002). There was no relationship demonstrated among the patients with dengue disease for age, sex, race, educational level, or type of occupation [24]. However, in one study conducted in the Klang Valley, foreign workers represented a higher proportion of people with acute DF compared with those without dengue disease (10·5% *vs* 3·5%) [25].

Regional studies conducted throughout Malaysia showed that the amount of rainfall, temperature, and humidity were all directly linked to dengue disease outbreaks [11], [13], [24], [26], [27]. Peak months for reported dengue disease cases tended to cluster around January to March and June to November (i.e., mostly during the two monsoon seasons of high rainfall) [12], [13]. However, national and local monthly surveillance demonstrated that dengue disease can occur all year round [12]. Several geographical monitoring and modelling studies have demonstrated that the increasing urbanization in Malaysia was a major risk factor for the recent rise in dengue disease incidence in the country [26], [28]–[30].

### Regional epidemiology

For 2007, dengue disease incidence data were available for all 14 Malaysian states. Regionally, the west peninsular states of Malaysia were most affected by dengue disease. Incidence rates in Kuala Lumpur, Selangor, Kelantan, and Penang areas were 455·7, 320·3, 224·5, and 204·5 per 100,000 population, respectively; with the exception of Pahang (179·1 per 100,000 population) the remaining states were less affected (<140 per 100,000 population) and incidence rates in south peninsular area (Malacca and Johor) and the Borneo states were <105 per 100,000 population [3]. In 2008, 63% of the national total of reported dengue disease cases occurred in the Klang Valley, which includes the state of Selangor and the Federal Territory of Kuala Lumpur [31].

Regional epidemiological data from Kelantan in 2003 [32] and Hulu Langat in 2003–2008 suggested the same pattern of increasing incidence of dengue disease as was reported for national data [33]. Hospital admission data for 2000–2003 in the Klang Valley, Selangor, were similar to national patterns [34].

### Seroprevalence

In a national cross-sectional study conducted in 2008, 91.6% of adults aged 35–74 years were DENV seropositive and the rate increased with age (from 80% in 35–44-year-olds to 94% in 55–74-year-olds) [35]. In a small, cross-sectional study conducted in Puchong in 2000–2001, the overall seropositive rate was 76.5%, rising from 33% in those aged <20 years to 100% in those aged >60 years [36]. Seroprevalence was reported to be 11% and 14% in two national cross-sectional studies in schoolchildren aged 7–18 years [28], [30].

### DENV serotype distribution

**Figure 4:**
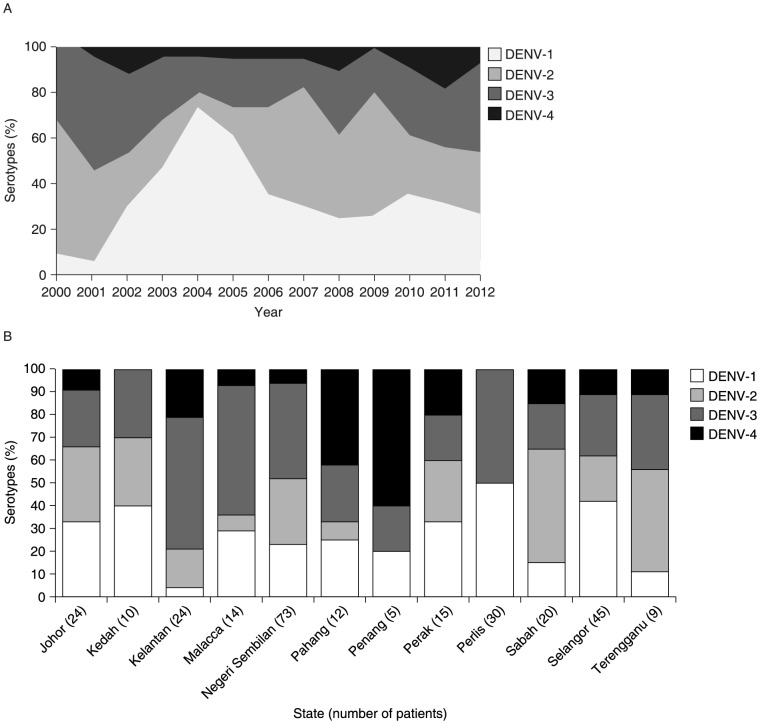
Serotype distribution in Malaysia. (A) National data 2000–2012 [3], [12]. (B) 2012 data (to 12 July 2012) by state* via the Sentinel Surveillance System [12]. *One patient in Sarawak was infected with DENV-1 (not shown on graph). National data (A) demonstrated that the dominant DENV serotypes circulating in the country changed continually throughout the period of 2000 to 2011. DENV-4 was less prevalent than the other three serotypes and constituted <20% of the serotypes for all years. Regional data from 1 January 2012 to 12 July 2012 reported via the Sentinel Surveillance System (B) showed a heterogeneous distribution of DENV serotypes in the separate states. DENV, dengue virus.

National data demonstrated that the dominant DENV serotypes circulating in the country changed continually during 2000–2011, from DENV-2 in 2000, to DENV-3 in 2001–2002, DENV-1 in 2003–2005, DENV-2 in 2006–2009, and DENV-1 in 2010–2011 (**Figure 4A**) [3], [12]. DENV-4 was less prevalent than the other three serotypes and constituted <20% of the serotypes for all years. A similar pattern of changing serotypes over time was reported from hospitalized patients in the east coast of peninsular Malaysia in 2005–2009 [37]. However, the predominant serotypes did not exactly coincide with those from the national data. DENV-1 predominated in 2005, DENV-1 and DENV-3 in 2006, DENV-1 and DENV-2 in 2007, and DENV-3 in 2008 and 2009.

Interestingly, the latest regional data from 1 January 2012 to 12 July 2012 reported in the Sentinel Surveillance System showed a heterogeneous distribution of DENV serotypes in the separate states (**Figure 4B**) [12]. DENV-1 predominated in four states and was equally dominant in two states, DENV-2 dominated in two states and was equally dominant in one state, DENV-3 dominated in three states and was equally dominant in one state, and DENV-4 dominated in two states. DENV-1 and -2 had equally dominant distributions in one state as did DENV-1 and -3.

## Discussion

### Epidemiology

This article provides a comprehensive overview of the evolving epidemiology of dengue disease in Malaysia over the period 2000–2012, despite there being some gaps in the information. As expected, the national data collated for our study and as reported recently by Mia et al. [38] show similar national trends of dengue disease incidence rates and deaths in Malaysia from 2000 to 2010. While our analysis supports their conclusions of an overall increase in the annual number of reported cases and deaths over the decade, we would propose that the data gathered from the second half of the decade show that the overall incidence seems to have stabilized at consistently high levels over that period. This pattern of incidence is specific to Malaysia and not observed in other countries in the Western Pacific region [3], [9], [12], [13], [16]–[23], [39]. Furthermore, we have incorporated 2011 data that reveal a sharp decline in incidence compared with previous years. As dengue disease is known to be unpredictable with respect to its incidence in any one year [3], it is difficult to draw any firm conclusions from the decline in incidence reported in 2011 [12]. Whilst annual changes in the level of under-reporting in any passive surveillance system may account for a reduction in the number of reported cases, the recent implementation of new action measures against dengue disease by the Malaysian MoH in 2009 may also be a factor. The measures include a strategic plan covering the years 2009–2013 [31], introducing clear treatment guidelines in 2010 [9]. In future, a new Sentinel Surveillance System in 2012 in which samples were collected from 40 sentinel sites (hospitals and health clinics) representing all states in Malaysia and sent to the nearest Public Health Laboratory for testing, and a protocol for sampling for virological confirmation and serotype assessment will ensure that the data collected are regionally representative and will contribute to future improvements in the knowledge of regional serotype circulation across Malaysia.

Towards the end of the 20th century and into the 21st century, the peak age group for reported dengue disease cases has been predominantly young adults. There was a slow increase in seroprevalence rates (slower than in other countries) [28], [30], [35], [36], consistent with the peak age incidence for clinically apparent dengue disease in young adults. These findings indicate a high level of DENV exposure in the community and show a different distribution to that observed in some other countries in the Asia–Pacific region, where seroprevalence is high in children, for example Thailand [40], [41], Vietnam [42], [43], and Indonesia [44]. However, Singapore has an even lower age-stratified seroprevalence in children than Malaysia [45] and has a higher peak age incidence for clinically apparent dengue disease [46].

There was a male predominance in the incidence data of reported dengue disease for the period 2000–2011 [12], [16], although the reasons for this are not clear and the differences between the sexes may be an artefact. Possible reasons for a reduced incidence of dengue disease in females include adult women being less likely than men to seek health care and relying on outpatient clinics and traditional remedies, as has been reported in Singapore [47], and occupational and socio-economic issues.

Up to the year 2000, most cases of dengue disease (70–80%) were reported in the urban population, particularly in working- and school-age groups [48], and this link to urbanization has continued into the 21st century [26], [28]–[30]. Increased urbanization is recognized as a major risk factor for the recent dramatic rise in dengue disease incidence in Malaysia [14]. Other identified risk factors for dengue disease mainly related to lack of personal protection against mosquito-borne infection [24] and factors promoting increased breeding areas for the vector mosquitoes (i.e., increased rainfall, temperature, and humidity) [11], [13], [24], [26], [27]. Modelling studies [26], [27] may help to predict outbreaks and direct the action required to reduce the number of mosquito breeding sites in order to reduce the risk of contracting dengue disease.

### DENV serotype distribution

Our analysis reveals that all four DENV serotypes were found to be co-circulating in Malaysia during the period 2000–2012, although the predominant serotypes varied over time, both nationally and within the individual states [3], [12], [37]. Similar patterns of distribution and changes in predominant circulating serotypes were reported by Mia et al. [38]. Over the past few decades, major dengue disease outbreaks occurred in a cyclical pattern, involving mainly DENV-1, DENV-2, and DENV-3 serotypes [7], although data on circulating DENV serotypes were sparse prior to 2000. Recent Malaysian MoH data from the Sentinel Surveillance System provide a more robust information source and 2012 data showed a heterogeneous distribution of the DENV serotypes in the separate states [12]. It can be speculated that changes in predominant circulating serotypes may have implications for the incidence and severity of dengue disease, as major outbreaks tend to follow the switching of DENV serotypes in the population [7]. The relationship between infection with DENV serotypes and the occurrence of different forms of the disease is multi-factorial and not fully understood. However, high dengue disease endemicity and the co-circulation of multiple DENV serotypes indicate that a high proportion of the population is at risk of severe dengue disease.

### Epidemiological knowledge gaps

From this analysis, it is clear that recent improvements in the national surveillance system have contributed to an improvement in epidemiological knowledge in Malaysia. Nevertheless, some gaps in epidemiological knowledge still exist; for example, there is a lack of data on age-stratified seroprevalence, long-term regional incidence and serotype distribution, relationships between age and disease severity, and hospital admissions. Furthermore, the national surveillance data are likely to have underestimated the true incidence of the disease in Malaysia owing to the use of a passive reporting system and low levels of reporting from private or primary care [10]. In addition, further investigations are required to examine the reasons for epidemiological patterns that appear to be specific to Malaysia, such as our hypothesis that the data collected show the incidence to have stabilised in Malaysia over the latter part of the review period, and the low age-related seroprevalence rate observed relative to other countries in the Western Pacific region.

### Limitations and strengths of the review

This literature survey and analysis reveals the changing epidemiology of dengue disease in Malaysia over the period 2000–2012 and demonstrates the consistency of the data collected. The substantial number of articles screened to identify relevant publications (over 200) and the comprehensive data extraction method used to capture the data both add strength to the results of this study.

However, there are limitations to this study, including the epidemiological knowledge gaps discussed above, which make it difficult to compare the epidemiology of the disease over time and between different geographical areas and different age groups. Furthermore, the data in this study were largely assessed based on national surveillance systems and are therefore subject to the limitations inherent to passive surveillance data, such as under-reporting, misreporting, and reporting biases. Passive reporting is clearly important for the identification of disease patterns and to guide vector control measures, but it may not be enough by itself to monitor progress in dengue disease control. The WHO 2011 revision of its 2009 dengue disease guidance [49] recommends that countries need to supplement their passive surveillance systems with sentinel and active surveillance programmes to determine accurately the burden of dengue disease.

### Conclusions

Dengue disease remains a major public health concern in Malaysia and is likely to remain endemic for a long time. There has been an increase in the incidence of all forms of dengue disease over 2000–2012. The predominant age group for dengue disease was young adults. Outbreaks tend to follow changes in predominant circulating DENV serotypes. Increasing levels of rainfall, humidity, temperature, and urbanization are also risk factors for outbreaks.

Malaysia has instituted improvements to its surveillance system that will, if continued and followed vigorously over the next few years, enable us to understand the true burden of dengue disease and to monitor the impact of dengue disease control measures in the future.

## Supporting Information

Table S1 Evidence table for citations fulfilling the inclusion and exclusion criteria for the literature review (n = 28).
(PDF)

Table S2 Dengue disease cases and incidence in Malaysia: national data.
(PDF)

Table S3 Dengue-related deaths and case-fatality rates in Malaysia: national data.
(PDF)

Checklist S1 PRISMA 2009 checklist.
(PDF)
