# Supplementary material for: Epidemiology of Dengue Disease in Malaysia (2000–2012): A Systematic Literature Review
Source: PLoS Negl Trop Dis. 2014 Nov 6;8(11):e3159. doi: 10.1371/journal.pntd.0003159 (PMC4222702; doi:10.1371/journal.pntd.0003159)
Supplement: Table S1 — Evidence table for citations fulfilling the inclusion and exclusion criteria for the literature review (n = 28). (PDF) [file pntd.0003159.s001.pdf]

Table S1. Evidence table for citations fulfilling the inclusion and exclusion criteria for the literature review (n=28).

Citation	Region/geographical area	Study design	Data period: Date range/year	If study:		Summary of data presentation or results/conclusion
				No. patients/ population studied [M:F]	Age range	
WHO, 2012 (2)	Nationwide data	WPRO surveillance data	2009			Online data tables.
Yusoff et al., 2008 (3)	Nationwide data	WPRO surveillance data	2000–2007			48,846 cases and 98 deaths were reported in 2007 (approximately 180 cases/100,000 population). The case-fatality rate was 3.2% in 2007. The highest proportion of cases affects patients between 20-24 years old. Kuala Lumpur and neighboring states reported the highest incidence rate. All four serotypes are present in the country.
MOH, 2010 (9)	Nationwide data	Ministry of Health surveillance data	2000–2007			The epidemiology review in these guidelines provides data for the number of dengue cases in Malaysia (1995–2007) and the incidence rate, number of deaths and case fatality rate by age group (1999–2007).The data show increasing numbers of DF/ DHF cases. The case fatality rates for DF/ DHF however remain below 0.3% since 2002. Most of the dengue cases were reported from urban areas (70 – 80%).

Citation	Region/geographical area	Study design	Data period: Date range/year	If study:		Summary of data presentation or results/conclusion
				No. patients/ population studied [M:F]	Age range	
Rohani et al., 2011 (11)	Kuala Lumpur, Pahang, Kedah, Johor	Dengue outbreak modelling study. A field study was conducted to collect and analyse various parameters to model dengue transmission and outbreak. The number of notified dengue cases reported in each locality during the study period was obtained from the local health authorities.	2007–2009	N/A	N/A	Previous week rainfall plays a significant role in increasing the mosquito population, followed by maximum humidity and temperature. The secondary data of rainfall, temperature and humidity provided by the meteorological department showed an insignificant relationship with the mosquito population compared to the primary data recorded by the researchers.
MOH., 2012 (12)	Nationwide data	Ministry of Health surveillance data				Online data tables.
WHO, 2012 (13)	Nationwide data	WPRO surveillance data	1990–2006			Data on current climate-sensitive disease burdens. Reported dengue cases by month and by morbidity week.
Anker et al., 2011 (16)	Malaysia	Ministry of Health surveillance data	2000–2008	M:F ratios reported by year	N/A	Reported number of incident dengue cases by age and sex for six countries in Asia. Data for Malaysia (1997–2008) were available by both sex and age-group separately but not in a form stratified for both. The majority of reported cases were persons > 15 years of age. The majority of reported cases were in men, For the two years for which regional data were available, each state consistently reported more cases in men (overall: 59%and 62% in 2007 and 2008, respectively).
Arima et al., 2011 (17)	Malaysia	Ministry of Health surveillance data	2010	46171	N/A	Dengue surveillance data for 2010 provided by Ministry of Health reported a total 46171 cases (134 fatal). While the number of cases fluctuated throughout the year, the peak (n = 1159 cases) was reported during August. While all four serotypes circulated, the predominant serotype identified was DENV-1, followed by

Citation	Region/geographical area	Study design	Data period: Date range/year	If study:		Summary of data presentation or results/conclusion
				No. patients/ population studied [M:F]	Age range	
						DENV-3 and DENV-2.
WHO, 2008 (18)	Nationwide data	WPRO surveillance data	2000–2004			This book combines information from the WHO South-East Asia and Western Pacific regions providing a single source of information on public health issues across the 48 countries and areas that form the Asia Pacific Region. In a review of the epidemiology od dengue in selected countries of the Region, the book provides data on the number of reported dengue cases and case-fatality rates in Malaysia, 1991–2004.
WHO, 2009 (19)	Nationwide data	WPRO surveillance data	2006–2008			A profile of health in WPRO countries. In 2007, 48 846 cases of dengue were reported: 46 095 (94.4%) were dengue fever and 2720(5.6%) dengue haemorrhagic fever. The dengue incidence rate was 179.2 per 100 000 population, compared with 144.7 per 100 000 population in 2006. Kuala Lumpur had the highest incidence rate, followed by Selangor, Kelantan and Penang.
WHO, 2011 (20)	Nationwide data	WPRO surveillance data	2010			A profile of health in WPRO countries.. In 2010, the number of dengue cases reported increased to 46 171 cases or an IR of 163.44 cases per 100 000 population. In 2010, Selangor had the highest IR, at 309 cases per 100 000 population, followed by Wilayah Persekutuan Kuala Lumpur-Putrajaya, with 250; Kelantan, with 213; Melaka, with 189; and Sarawak, with 166. The number of dengue deaths also

Citation	Region/geographical area	Study design	Data period: Date range/year	If study: No. patients/ population studied [M:F]	If study: Age range	Summary of data presentation or results/conclusion
						increased, from 88 (case fatality rate = 0.21%) in 2009 to 134 (0.29%) in 2010.
Dom et al., 2010 (21)	Subang Jaya, Selangor	Spatial and temporal mapping study. Surveillance data used to enhance spatial modelling using temporally defined indices.	2005–2008			Measurement of the three temporal risk indices found that the mean frequency index was < 1 (1 indicates that dengue will occur every week throughout the year). The mean value of duration index was 1.66 (equating to a mean epidemic duration of about 2 weeks).. The mean intensity index of 5.41 signified that about 5–6 people/1000 population would be infected by dengue fever during an epidemic period.
WHO, 2013 (22)	Nationwide data	WPRO surveillance data	2000–2010			Online data tables.
WHO, 2011 (23)	Nationwide data	WHO survey	2009-11			Regular dengue situation updates are made available by the WHO WPRO based on officially reported national surveillance data.
Norli et al., 2008 (24)	Johor Bahru	Case–control study to examine distribution of dengue disease.	2006	77 cases [48.1%:51.9%]; 77 controls [46.8%:53.2%]	Mean: 31.62 years (cases); 31.92 years (controls)	Time-series analysis indicated that the cases of dengue illness were related to changes in the minimum temperature. Multivariate analysis indicated that those who were unmarried, not wearing long sleeve clothes and whose homes were not protected with screen windows were at greatest risk of contracting dengue.

Citation	Region/geographical area	Study design	Data period: Date range/year	If study: No. patients/ population studied [M:F]	If study: Age range	Summary of data presentation or results/conclusion
Mustafa et al., 2010 (25)	Four primary healthcare centres (Poliklinik Seksyen 7, Shah Alam; Poliklinik Kelana Jaya, Petaling Jaya; Poliklinik Sg. Buloh; Poliklinik Jinjang) in the Klang valley	Prospective evaluation of commercial dengue NS1 antigen capture ELISA kit with capture of the epidemiological and clinical features of patients recruited for the study.	2006–2009	558 [344:214]	1.5–76 years; Mean: 26 years	Based on laboratory test, 190 patients were categorized as acute dengue fever, 86 as recent dengue and 282 as non-dengue febrile illnesses. The mean age of patients in the dengue fever group was significantly younger in comparison with patients in the non-dengue group. No significant difference between the two groups with respect to sex but there was significant ethnic difference with foreign workers representing a higher proportion in the dengue fever group.
Seng et al., 2005 (26)	Johor State	Geographical weighted regression (GWR) analysis of relationship between population and dengue fever prevalence.	2004	N/A	N/A	Geostatistical modelling, analysis and mapping revealed a strong positive spatial association between dengue fever prevalence and population distribution in the Johor State. The study also identified that the DENV incubation period is 15 days. Dengue transmission was limited at 200m and 20 days and mainly involved household transmission (a contagious pattern).
Ibrahim et al., 2011 (27)	Hulu Langat, Selangor	Modelling spread of dengue outbreak using data acquired from Selangor State Health Department.	2007–2011	21436	N/A	The data set consisted of 227 weekly measurements of total rainfall, mean temperature and dengue cases in Hulu Langat (2007–2011). A simulation of the system dynamic was to predict the spread of dengue outbreak. Data analysis showed that dengue cases can be predicted accurately using the two main variables – rainfall and temperature.

Citation	Region/geographical area	Study design	Data period: Date range/year	If study: No. patients/ population studied [M:F]	If study: Age range	Summary of data presentation or results/conclusion
Tiong et al., 2010 (28)	16 areas throughout Malaysia	Seroprevalence study to examine the relationship between land cover features and prevalence of dengue.	N/A	1410	7–18 years	The presence of dengue-specific IgG was taken to be indicative of previous exposure to dengue. 11% of the study population was seropositive for dengue. Seropositivity ranged from 0–25.86%. Dengue prevalence had an inverse relationship with vegetation but correlated directly with area of land development. Sites with >10% prevalence comprised primarily of residential areas with closely laid out terrace houses.
Shafie et al., 2011 (29)	Georgetown, Penang Island	Development of spatial model for areas with high dengue risk and the prediction of risk for DF and DHF utilising the data on dengue incidences in Georgetown area.	2000–2004	N/A	N/A	Logistic regression analysis showed only 10/16 variables significantly influence the probability of the incidence of DF and DHF at the 0.05 level. The accuracy of the resulting model is 70.3%. Using these variables a spatial model equation was developed and used to calculated and map the incidence of DF and DHF in the study area, indicating areas with the highest risk.
Abu Baker et al., 2011 (30)	Malaysia	Seroepidemiologic study and geospatial analyses	N/A	1800 schoolchildren; ~500 Orang Asli population	7–18 years (children); adults (Orang Asli)	Study confirmed that dengue is present throughout Malaysia. Approximately 14% (range: <1% to ~25%) of school children have been exposed to dengue by the age of 13. Among the Orang Asli populations the percentage of samples positive for dengue was at ~17% (range: <3% to ~51%). Geospatial analyses suggested a correlation between the prevalence of dengue and land development.
MOH., 2009 (31)	Nationwide data	Ministry of Health surveillance data				Report includes a situation analysis for 2008 when 49,335 cases with 113 deaths were reported (the highest number of cases ever reported in Malaysia at that time). Most (63%)

Citation	Region/geographical area	Study design	Data period: Date range/year	If study: No. patients/ population studied [M:F]	If study: Age range	Summary of data presentation or results/conclusion
						of these cases occurred in the Klang Valley (which incorporates the state of Selangor and Federal Territory of Kuala Lumpur). The majority of reported cases occurred in urban areas.
Hussin et al., 2005 (32)	Kota Bharu, Kelantan, Malaysia	Retrospective data review of all dengue cases notified to Kota Bharu district Health Office, Kelantan, Malaysia (1998 to 2003).	1998–2003	4716 [55.2%:44.8%]	Mean: 27.8 years	Between 1997 and 2003, 4,716 dengue cases were notified; DF: 4,476 (94.9%), DHF: 240 (5.1%). The months with the three highest numbers of cases were during the rainy season in Kelantan (December, January and February). The study also reported the number of DF and DHF cases by year, showing that the incidence of dengue generally increased in Kota Bharu and is comparable to that across Malaysia.
Mazrura et al., 2010 (33)	Urban and rural districts: Seremban, Ledang and Hula Langat river basin	2003- 2008 surveillance data. Research methodologies included site visits, review of surveillance data and mapping on Aedes population, dengue cases and climate variability.	2003–2008	N/A	N/A	The highest incidence of dengue cases recorded in Seremban was in 2003. Since 2005, cases decreased by 20-40%. For Hulu Langat, the dengue cases increased sharply in 2005 and remained high until 2008. The mosquito survey showed a sudden increased of the Aedes population, from 22.7% (dry season) to 53.0% (rainy season).
Jamaiah et al., 2005 (34)	Hospital Tengku Ampuan Rahimah, Klang, Selangor	Risk factor comparison study using data acquired from the database of the hospital record department.	1999–2003	6577 (DF) [3874:2703]; 857 (DHF) [445:412]	<1–>12 years	A total of 6,577 DF cases and 857 DHF cases of dengue hemorrhagic fever were reported (1999–2003). Although there was a slight decrease in reported cases in 2001, overall DF cases increased dramatically from 2001; most cases occurred in 2003. Highest DF incidence was in Malay males >12 years of age.

Citation	Region/geographical area	Study design	Data period: Date range/year	If study: No. patients/ population studied [M:F]	If study: Age range	Summary of data presentation or results/conclusion
Muhammad et al., 2011 (35)	Nationwide (representative cohort)	Cross-sectional seroepidemiology cohort study to determine the prevalence of dengue antibodies in the Malaysian adult population.	2008	1000 [400 (40%):600 (60%)]	35–74 years	91.6% subjects were dengue IgG seropositive (375 males and 541females). Age has a significant association with seropositivity (seroprevalence increased with every 10 year increase in age). Sex and ethnicity had no effect. Similar seroprevalence rates were observed in both urban and rural samples.
Chen et al., 2003 (36)	Puchong	Cross-sectional seroprevalence study	2000–2001	85 [46 (54.1%): 39 (45.9%)]	5–71 years (Mean: 42.8 years)	Letter to the Editor of the Medical Journal of Malaysia reported a study with PanBio (PanBio Pty Ltd, Australia) dengue indirect IgG enzyme-linked immunosorbent assay kit that confirmed increasing seroconversion rate with increasing age. The overall seropositive rate was 76.5% (<20 years old: 33%; 21-40 years: 63%; 41-60 years: 88%; 61-80 years: 100%).
Vinomarlini et al., 2011 (37)	East coast of peninsula Malaysia: Kelantan, Pahang, Terengganu	Open, prospective serotype prevalence study.	2005–2009	488 serum and blood samples from hospitalized patients	>10–>61 years	Overall 134 samples (37.5%) were positive for DENV infection. The prevailing dengue serotypes were determined using a real time polymerase chain reaction assay (RT-PCR). All 4 DENV serotypes circulated during the study period.. The predominant serotypes circulating in 2005–6 were DENV-1 and DENV-3; DENV-1 and DENV-2 were predominant in 2007; DENV-3 was the predominant serotype in 2008–9.
