# Supplementary material for: Epidemiology of Dengue Disease in Malaysia (2000–2012): A Systematic Literature Review
Source: PLoS Negl Trop Dis. 2014 Nov 6;8(11):e3159. doi: 10.1371/journal.pntd.0003159 (PMC4222702; doi:10.1371/journal.pntd.0003159)
Supplement: Table S2 — Dengue disease cases and incidence in Malaysia: national data. (PDF) [file pntd.0003159.s002.pdf]

Supplementary Table S2. Dengue disease cases and incidence in Malaysia: national data.

Year Population (1000s)	Cases reported (n) (All)	Laboratory-confirmed cases (%) (All)	Cases reported (n) (DF)	Cases reported (n) (DHF/DSS)	Incidence per 100,000 population (All)	Source of data First author, year[Ref]
2000 23,494.9	7103	52.4	6692	411	31.6	Anker 2011[16], WHO 2012[13], Dom 2010[21], MOH 2010[9], WHO 2008[3], WHO 2008[18], MOH 2012[12]
2001 24,123.4	16,368	53.0	15,446	922	72.1	Anker 2011[16], WHO 2012[13], Dom 2010[21], MOH 20109, WHO 2008[3], WHO 2008[18], MOH 2012[12]
2002 24,727.1	32,767	47.3	30,807	1960	133.5	Anker 2011[16], WHO 2012[13], Dom 2010[21], MOH 2010[9], WHO 2008[3], WHO 2008[18], WHO (multiple years)*, MOH 2012[12]
2003 25,320.0	31,545	49.0	30,221	1324	125.9	Anker 2011[16], WHO 2012[13], Dom 2010[21], MOH 2010[9], WHO 2008[3], WHO 2008[18], MOH 2012[12]
2004 25,905.1	33,895	39.6	32,422	1473	132.5	Anker 2011[16], WHO 2012[13], Dom 2010[21], MOH 2010[9], WHO 2008[3], WHO 2008[18], MOH 2012[12]
2005 26,476.9	39,686	42.7	37,646	2040	150.6	Anker 2011[16], WHO 2012[13], Dom 2010[21], MOH 2010[9], WHO 2008[3], MOH 2012[12]
2006 26,831.5	38,556	47.3	36,462	2094	148.3	Arima 2011[17], Anker 2011[16], WHO 2012[13], MOH 2010[9], WHO 2008[3], WHO 2009[19], MOH 2012[12]
2007 27,186.0	48,846	48.7	45,856	2990	180.0	Arima 2011[17], Anker 2011[16], MOH 2010[9], WHO 2008[3], WHO 2009[19], MOH 2012[12]

Year Population (1000s)	Cases reported (n) (All)	Laboratory-confirmed cases (%) (All)	Cases reported (n) (DF)	Cases reported (n) (DHF/DSS)	Incidence per 100,000 population (All)	Source of data First author, year[Ref]
2008 27,540.5	49,335		46,517	2818	178.0	Arima 2011[17], Anker 2011[16], MOH 2012[12]
2009 27,895.3	41,486		38,749	2737	147.0	Arima 2011[17], WHO 2011[23], WHO 2012[2], MOH 2012[12]
2010 28,250.5	46,171		42,140	4031	159.7	Arima 2011[17], WHO 2011[20], WHO 2011[23], MOH 2012[12]
2011 28,552.7	19,884		18,466	1418	69.6	MOH 2012[12]
2011 (to 31/1/11)	2084					WHO 2012[2]
2012 (to 31/1/12) 28,854.9	1868					WHO 2012[2]

DF, dengue fever; DHF, dengue haemorrhagic fever; DSS, dengue shock syndrome. *World Health Organization. Number of reported cases of dengue fever and dengue hemorrhagic fever (DF/DHF) in the Western Pacific Region, by country. India: World Health Organization (several years).
