# Supplementary material for: Epidemiology of Dengue Disease in Malaysia (2000–2012): A Systematic Literature Review
Source: PLoS Negl Trop Dis. 2014 Nov 6;8(11):e3159. doi: 10.1371/journal.pntd.0003159 (PMC4222702; doi:10.1371/journal.pntd.0003159)
Supplement: Table S3 — Dengue-related deaths and case-fatality rates in Malaysia: national data. (PDF) [file pntd.0003159.s003.pdf]

Supplementary Table S3. Dengue-related deaths and case-fatality rates in Malaysia: national data.

Year	Death cases (n)			Case fatality rate (%)			Source of data<br>First author, year[Ref]
	DF	DHF/DSS	All	DF	DHF/DSS	All	
2000	0	45	45	0	10.9	0.63	Dom 2010[21], MOH 2010[9], WHO 2008[3], MOH 2012[12]
2001	0	50	50	0	5.4	0.31	Dom 2010[21], MOH 2010[9], WHO 2008[3], MOH 2012[12]
2002	0	99	99	0	5.1	0.30	Dom 2010[21], MOH 2010[9], WHO 2008[3], MOH 2012[12]
2003	0	72	72	0	5.4	0.23	Dom 2010[21], MOH 2010[9], WHO 2008[3], MOH 2012[12]
2004	0	102	102	0	6.9	0.30	Dom 2010[21], MOH 2010[9], WHO 2008[3], MOH 2012[12]
2005	0	107	107	0	5.2	0.27	Dom 2010[21], MOH 2010[9], WHO 2008[3], MOH 2012[12]
2006	0	92	92	0	4.4	0.24	Dom 2010[21], MOH 2010[9], WHO 2008[3], MOH 2012[12]
2007	0	98	98	0	3.3	0.20	Dom 2010[21], MOH 2010[9], WHO 2008[3], MOH 2012[12]
2008	0	112	112	0	4.0	0.23	Arima 2011[17], MOH 2012[12]
2009	0	88	88	0	3.2	0.21	Arima 2011[17], MOH 2012[12]
2010	0	134	134	0	3.3	0.29	Arima 2011[17], MOH 2012[12]
2011	0	36	36	0	2.5	0.18	MOH 2012[12]

DF, dengue fever; DHF, dengue haemorrhagic fever; DSS, dengue shock syndrome.
